# The importance of patient characteristics, operators, and image quality for the accuracy of heart failure diagnosis by general practitioners using handheld ultrasound devices

**DOI:** 10.1093/ehjimp/qyad047

**Published:** 2023-12-29

**Authors:** Malgorzata Izabela Magelssen, Anna Katarina Hjorth-Hansen, Garrett Newton Andersen, Torbjørn Graven, Jens Olaf Kleinau, Kyrre Skjetne, Lasse Lovstakken, Havard Dalen, Ole Christian Mjølstad

**Affiliations:** Department of Circulation and Medical Imaging, Norwegian University of Science and Technology, Prinsesse Kristinas gt 3, Akutten og Hjerte-lunge-senteret, 7491Trondheim, Norway; Clinic of Cardiology, St. Olavs Hospital, Trondheim University Hospital, Prinsesse Kristinas gt 3, Akutten og Hjerte-lunge-senteret, 7491 Trondheim, Norway; Department of Circulation and Medical Imaging, Norwegian University of Science and Technology, Prinsesse Kristinas gt 3, Akutten og Hjerte-lunge-senteret, 7491Trondheim, Norway; Department of Internal Medicine, Levanger Hospital, Nord-Trøndelag Hospital Trust, Kirkegate 2, 7600 Levanger, Norway; Department of Internal Medicine, Levanger Hospital, Nord-Trøndelag Hospital Trust, Kirkegate 2, 7600 Levanger, Norway; Department of Internal Medicine, Levanger Hospital, Nord-Trøndelag Hospital Trust, Kirkegate 2, 7600 Levanger, Norway; Department of Internal Medicine, Levanger Hospital, Nord-Trøndelag Hospital Trust, Kirkegate 2, 7600 Levanger, Norway; Department of Internal Medicine, Levanger Hospital, Nord-Trøndelag Hospital Trust, Kirkegate 2, 7600 Levanger, Norway; Department of Circulation and Medical Imaging, Norwegian University of Science and Technology, Prinsesse Kristinas gt 3, Akutten og Hjerte-lunge-senteret, 7491Trondheim, Norway; Department of Circulation and Medical Imaging, Norwegian University of Science and Technology, Prinsesse Kristinas gt 3, Akutten og Hjerte-lunge-senteret, 7491Trondheim, Norway; Clinic of Cardiology, St. Olavs Hospital, Trondheim University Hospital, Prinsesse Kristinas gt 3, Akutten og Hjerte-lunge-senteret, 7491 Trondheim, Norway; Department of Internal Medicine, Levanger Hospital, Nord-Trøndelag Hospital Trust, Kirkegate 2, 7600 Levanger, Norway; Department of Circulation and Medical Imaging, Norwegian University of Science and Technology, Prinsesse Kristinas gt 3, Akutten og Hjerte-lunge-senteret, 7491Trondheim, Norway; Clinic of Cardiology, St. Olavs Hospital, Trondheim University Hospital, Prinsesse Kristinas gt 3, Akutten og Hjerte-lunge-senteret, 7491 Trondheim, Norway

**Keywords:** handheld ultrasound device, decision-support software, ejection fraction, mitral annular plane systolic excursion, telemedicine, heart failure

## Abstract

**Aims:**

To evaluate whether the characteristics of patients, operators, and image quality could explain the accuracy of heart failure (HF) diagnostics by general practitioners (GPs) using handheld ultrasound devices (HUDs) with automatic decision-support software and telemedical support.

**Methods and results:**

Patients referred to an outpatient cardiac clinic due to symptoms indicating HF were examined by one of five GPs after dedicated training. In total, 166 patients were included [median (inter-quartile range) age 73 (63–78) years; mean ± standard deviation ejection fraction 53 ± 10%]. The GPs considered whether the patients had HF in four diagnostic steps: (i) clinical examination, (ii) adding focused cardiac HUD examination, (iii) adding automatic decision-support software measuring mitral annular plane systolic excursion (autoMAPSE) and ejection fraction (autoEF), and (iv) adding telemedical support. Overall, the characteristics of patients, operators, and image quality explained little of the diagnostic accuracy. Except for atrial fibrillation [lower accuracy for HUD alone and after adding autoEF (*P* < 0.05)], no patient characteristics influenced the accuracy. Some differences between operators were found after adding autoMAPSE (*P* < 0.05). Acquisition errors of the four-chamber view and a poor visualization of the mitral plane were associated with reduced accuracy after telemedical support (*P* < 0.05).

**Conclusion:**

The characteristics of patients, operators, and image quality explained just minor parts of the modest accuracy of GPs’ HF diagnostics using HUDs with and without decision-support software. Atrial fibrillation and not well-standardized recordings challenged the diagnostic accuracy. However, the accuracy was only modest in well-recorded images, indicating a need for refinement of the technology.

## Graphical Abstract

**Graphical Abstract:**
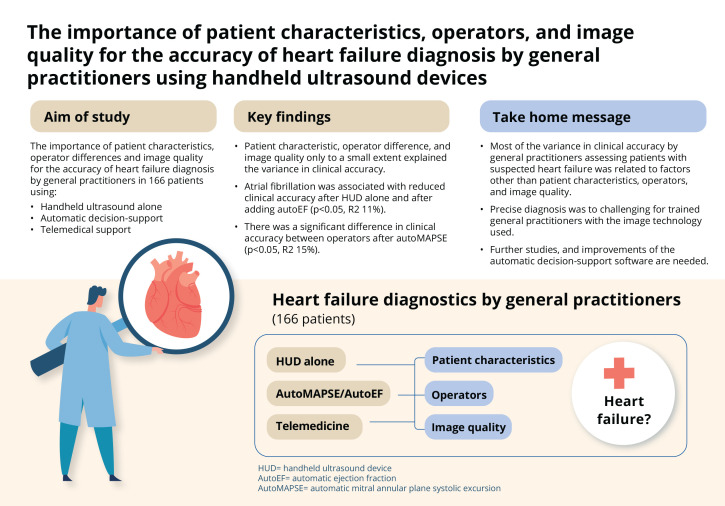

## Introduction

Heart failure (HF) has overlapping symptoms with comorbidities such as atrial fibrillation (AF), chronic obstructive pulmonary disease (COPD), and obesity.^1,2^ A thorough clinical examination may lead to a suspicion of HF, but an echocardiographic assessment with quantification of the left ventricle (LV) is the diagnostic cornerstone.^3^ Handheld ultrasound devices (HUDs) can be used by adequately trained inexperienced users to evaluate LV function.^4,5^ Automatic decision-support software for LV quantification and telemedical support is available on HUDs and can aid inexperienced users.^6–8^ In a recent publication, it was shown that the diagnostic accuracy of HUD examinations by inexperienced users was modestly improved by telemedical support, while automated analyses of ejection fraction (autoEF) and mitral annular plane systolic excursion (autoMAPSE) did not improve diagnostic accuracy.^9^ To the best of our knowledge, it is unclear how comorbidities, operator characteristics, and image quality influence the diagnostic accuracy of HUD examinations by inexperienced operators supported by decision-support software. Thus, we aimed to study the associations of patient characteristics, operators, and image quality with the diagnostic accuracy of general practitioners (GPs) enhancing their clinical evaluation with HUD examinations with and without automatic decision-support software and telemedical support in a population with symptoms suggestive of HF.

## Methods

Comprehensive details of the study population and methodology were recently published.^9–11^

### Study population

Patients referred to the outpatient clinic at Levanger Hospital, Norway, with symptoms suggestive of HF were included from June 2018 to June 2020 with a 3-month pause due to the COVID-19 pandemic. Inclusion criteria were symptoms suggestive of HF, N-terminal pro-brain natriuretic peptide (NT-proBNP) ≥125 ng/L, age ≥18 years, and the ability to provide written consent.

Of 185 invited, 170 consented to participate. Four were excluded, and thus, 166 patients were included in the study.^9–11^

### Training

Six GPs inexperienced in diagnostic ultrasound were recruited by the municipality administration to participate in the study. One GP was relocated before the start of inclusions and did not participate. The GPs received two theoretical lectures by experts and 6 days of in-hospital training focusing on cardiac examinations by HUDs, supervised by two cardiology residents.^9–11^ Each GP performed on average 7 supervised examinations per day in addition to an average of 13 unsupervised examinations, summing up to the median [inter-quartile range (IQR)] of 46 (45–68) examinations prior to the start of the study.

### Study flow

**Figure 1:**
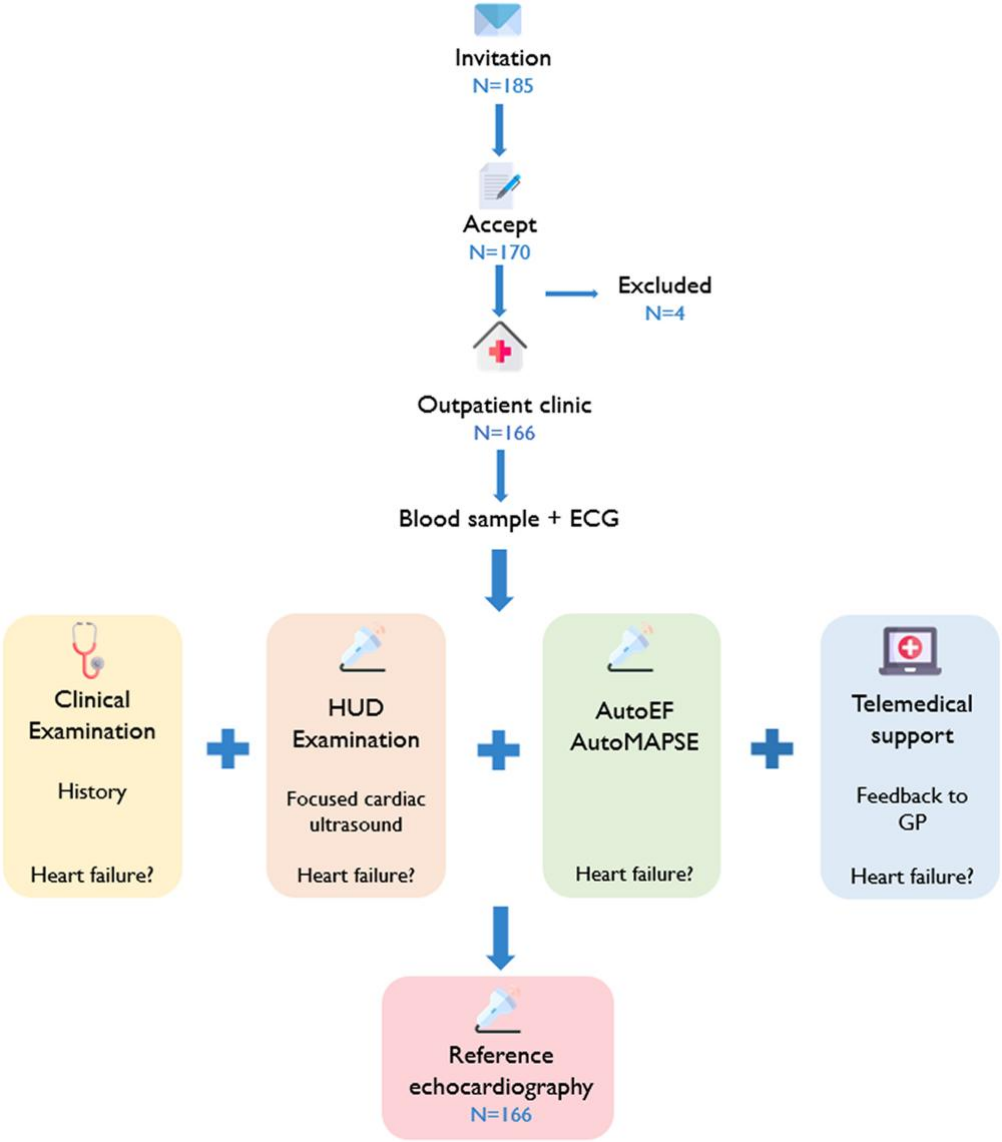
A flow of the study participants. The figure illustrates the flow of the study participants through the study.

Beyond the scope of this manuscript, experienced nurses additionally examined the patients by HUDs. The reference examinations by cardiologists included both echocardiography and HUD recordings.^10,11^ *Figure 1* shows the patient flow through the study. Blood samples were analysed for NT-proBNP, creatinine, sodium, potassium, and haemoglobin. Height, weight, blood pressure, and electrocardiogram (ECG) were recorded. On each inclusion day, one of five GPs examined each participant in several diagnostic steps. At each step, the GPs considered whether the patients had HF. First, they performed a standard clinical examination. Secondly, a cardiac ultrasound examination using a HUD was added. Thirdly, new HUD recordings were quantified using autoMAPSE and autoEF. Finally, the recorded images were automatically transferred to a cloud-based server (Tricify, Trice Imaging Inc., Del Mar, CA, USA) for analysis by one of two external cardiologists, St. Olavs Hospital, Trondheim, Norway. The external cardiologists provided feedback to the GPs in near real time.

Lastly, the patients were examined by one of five in-hospital cardiologists. Their diagnosis, based on the clinical assessment, laboratory results, and a comprehensive echocardiograpy, was used as a reference. The patients received treatment in accordance with the results from the reference examination. All operators were blinded to details from the other operators’ assessments.

### Cardiac ultrasound

The Vscan Extend (GE Healthcare, Horten, Norway) equipped with commercial software for autoEF (DiA Imaging Analysis Ltd, Be’er Sheva, Israel), research software for autoMAPSE (NTNU, Trondheim, Norway), and software for a secure transfer of image data for telemedicinal support (Trice Imaging) was used in the study. Comprehensive details of the software have been previously described.^6,7,12^ The autoEF application was revised during the study due to an error influencing calculations of LV volumes and ejection fraction (EF).

Based on visual assessment, the GPs categorized the LV function as normal, modestly, or severely reduced. The inferior caval vein was identified as dilated or not. Pathological amounts of pericardial or pleural effusion were noted.

AutoEF and autoMAPSE were launched in apical four-chamber view recordings and repeated in three recordings. LV EF was considered normal when ≥50% and MAPSE when >10 mm. The GPs could discard recordings if failure or miscalculations of the software were revealed.

The external cardiologists downloaded images from Tricefy to EchoPAC SWO (version 202, GE HealthCare). They had access to initial referrals but not to information from the clinical examination, blood samples, or ECG.

The reference echocardiography was performed according to current recommendations using high-end equipment (Vivid E9 or E95, GE Healthcare).^13^ EchoPAC SWO (version 202) was used for analyses.

The GPs’ initial diagnosis was based on patient history, symptoms, signs, and ECG findings. Due to laboratory delays, NT-proBNP results were not always available. In the following diagnostic steps, the evaluation of cardiac size and function was included in the decision-making process: first, by their own interpretation, then supported by the automated decision-support software, and lastly after telemedical feedback. The reference cardiologists categorized HF as HF with preserved EF (HFpEF; EF ≥50%), HF with mildly reduced EF (HFmrEF; EF 40–49%), or HF with reduced EF (HFrEF; EF <40%) based on the Simpson’s biplane calculations.^14^

### Patient characteristics

Ongoing AF was based on the recorded ECG. COPD was defined as not fully reversible airflow limitation documented in either the referrals or the hospital records. Body mass index (BMI; weight divided by height squared) was categorized according to the World Health Organization categorization as underweight (<18.5 kg/m^2^), normal (18.5–24.9 kg/m^2^), overweight (25–29.9 kg/m^2^), and obesity (≥30 kg/m^2^).^15^

### Operator characteristics

Data from each GP were analysed. To evaluate the importance of any learning effect, the study period was dichotomized to early or late according to the first and second half of the included patients for each GP.

### Image quality

Recordings with either of the automatic decision-support software applications were scored by one of the external cardiologists according to predefined categories (LV 4-chamber view, LV alignment, malposition of the apex, mitral annular assessment, and the number of LV segments with visible endocardium), with scores ranging from 1 (lowest) to 6 (highest).^11^ A mean score was calculated by averaging the scores from the categories. The average of the scores from autoEF and autoMAPSE recordings was used when evaluating the diagnostic accuracy after telemedical support.

### Statistics

Continuous data are presented as mean ± standard deviation (SD) if normally distributed and median (IQR) if skewed. Categorical data are expressed as numbers and frequencies (percentages). Associations of patient characteristics, operators, and image quality with the diagnostic accuracy were analysed using binary logistic regression and adjusted for relevant characteristics (EF, BMI, and image score). The analyses were performed at each diagnostic step (and separately for autoEF and autoMAPSE). Nagelkerke *R*^2^ was used as an indicator of the variance explained by the model. Differences between operators were analysed by using the *χ*^2^ test. The Kruskal–Wallis *H* test was used to compare the image scores between operators. The difference in the diagnostic accuracy between early and late study periods, as well as the difference before and after the autoEF software upgrade, was analysed using binary logistic regression.

Power estimations were performed for the main study only and based on the ability to detect a difference of 15% in correctly diagnosed HF patients compared with reference. Details have been recently published, but the sample of 104 originally needed was increased to 170 to account for a high number of normal findings and the revision of the autoEF software.^9^ A *P*-value <0.05 was considered statistically significant. Analyses were performed using SPSS statistics versions 27 and 28 (IBM Corporation, Armonk, NY, USA).

### Ethical considerations

The study was approved by the regional committee for medical and health research ethics (REK 2017/2054) and registered in the ClinicalTrial.gov database (identifier: NCT03547076). The study was performed in conformity with the Declaration of Helsinki.

## Results

### Study population

Baseline data have been previously published.^9–11^ Shortly, the median (IQR) age was 73 (63–78) years, and 47% were female. The BMI was ≥25 kg/m^2^ in 132 (80%) patients, of whom 59 (45%) were obese (BMI ≥30 kg/m^2^). Ongoing AF and COPD were present in 40 (24%) and 26 (16%) patients, respectively (*Table 1*). The reference cardiologist diagnosed HF in 28 (17%) patients.

**Table 1:** Baseline characteristics

Variable	Value
Age, years	73 (63–78)
Female sex, *n* (%)	78 (47)
Height, cm	172 ± 10
Weight, kg	85 ± 19
BMI, kg/m^2^	29 ± 5
Heart rate, bpm	77 ± 16
Systolic blood pressure, mmHg	150 ± 22
Diastolic blood pressure, mmHg	83 ± 11
NT-proBNP, ng/L	298 (65–780)
Creatinine, µmol/L	84 (73–97)
AF, *n* (%)	40 (24)
COPD, *n* (%)	26 (16)
Coronary artery disease, *n* (%)	19 (11)
Diabetes mellitus, *n* (%)	22 (14)
Hypertension, *n* (%)	60 (35)
Diuretics, *n* (%)	41 (25)
Beta-blocker, *n* (%)	51 (31)
Angiotensin-converting enzyme inhibitor, *n* (%)	32 (19)
EF, %	53 ± 10
MAPSE (mean of septum and anterolateral wall), mm	12 ± 3
Indexed left atrial end-systolic volume, mL/m^2^	42 ± 16

Values are given as mean ± SD or median (IQR), unless otherwise specified. Echocardiographic data are from reference examinations.

Of the 28 patients, 13 (46%) had HFpEF, 4 (14%) HFmrEF, and 11 (39%) HFrEF. Furthermore, 15 (54%) of the HF patients had ongoing AF, and 6 (21%) had COPD. The reference examination revealed normal LV function in 131 (79%) participants, modestly reduced LV function in 21 (13%), and severely reduced in 14 (8%).

### Patient characteristics

*Tables 2* and *3* show the diagnostic accuracy and its associations with patient characteristics. The results for the overall diagnostic accuracy of GPs at different diagnostic steps were recently published but are included here for comparison.^9^

**Table 2:** Diagnostic accuracy according to patient characteristics

	HUD alone	AutoMAPSE	AutoEF	Telemedicine
AF				
Correct	21 (53)	17 (43)	15 (38)	24 (60)
Incorrect	10 (25)	10 (25)	9 (23)	6 (15)
Uncertain	6 (15)	10 (25)	13 (33)	9 (23)
COPD				
Correct	15 (58)	12 (46)	12(46)	19 (73)
Incorrect	5 (19)	5 (19)	4 (15)	2 (8)
Uncertain	5 (19)	8 (31)	9 (35)	4 (15)
BMI ≥25 mg/kg^2^				
Correct	95 (72)	77 (58)	78 (59)	99 (75)
Incorrect	16 (12)	22 (17)	13 (10)	11 (8)
Uncertain	15 (11)	26 (20)	29 (22)	17 (13)
HFpEF^a^				
Correct	7 (54)	6 (46)	3 (23)	7 (54)
Incorrect	4 (31)	4 (31)	4 (31)	5 (38)
Uncertain	2 (15)	3 (23)	6 (46)	1 (8)
HFmrEF^a^				
Correct	3 (75)	3 (75)	3 (75)	3 (75)
Incorrect	0	0	0	0
Uncertain	1 (25)	1 (25)	0	1 (25)
HFrEF^a^				
Correct	9 (82)	8(73)	9 (82)	10 (91)
Incorrect	2 (18)	0	0	1 (9)
Uncertain	0	3 (27)	2 (18)	0

Data are given in numbers (percentages) of correct, incorrect, and uncertain diagnoses by GPs in subgroups of patients with the specified characteristics. Uncertain represents the GPs’ diagnostic uncertainty.

^a^Based on reference examinations.

**Table 3:** Associations of patient characteristics with diagnostic accuracy

	*n* with/*n* without characteristics	HUD alone *β* (*R*^2^)	AutoMAPSE *β* (*R*^2^)	AutoEF *β* (*R*^2^)	Telemedicine *β* (*R*^2^)
Patient characteristics					
AF	40 (24)/126 (76)	−1.4 (10)^a^	−0.7 (2.7)	−1.4 (10)^a^	−1.1 (5.2)
COPD	26 (16)/139 (84)	−0.8 (2)	−0.2 (0.2)	−0.5 (1)	0.09 (0)
BMI (≥25 kg/m^2^)	132 (80)/34 (20)	0.3 (0.3)	0.9 (4)	1.1 (5)	0.1 (0)
BMI (≥30 kg/m^2^)	59 (36)/107 (64)	0.1 (0)	−0.3 (1)	0.07 (0)	−0.7 (0)
EF ≥ 50%^b^	131 (79)/35 (21)	0.4 (4)	0.6 (1)	0.9 (3)	1.1 (5)
EF 40–49%^b^	21 (13)/145 (87)	−0.9 (2)	−1 (3)	−1.3 (4)	−1.2 (4)
EF < 40%^b^	14 (8)/152 (92)	−0.7 (1)	0.04 (0)	−0.3 (0)	−0.7 (1)

Data are given in numbers (percentages), beta coefficient, and *R*^2^. *β* represents a positive or negative association. *R*^2^ indicates the percentage of variance attributed to the characteristic.

^a^Statistically significant (*P* < 0.05).

^b^Based on reference echocardiography.

Ongoing AF was associated with reduced diagnostic accuracy at each diagnostic step, but the associations were significant only for HUD alone and after autoEF (*P* ≤ 0.007, both *R*^2^ = 10%). When adjusting ongoing AF for reference LV EF dichotomized using a cut-off LV EF of 50%, the associations remained significant with *P* ≤ 0.02, *R*^2^ = 10% for both HUD alone and autoEF. The combination of AF and the averaged image quality score for the four-chamber view recordings explained more of the variance in diagnostic accuracy after HUD alone (*P* = 0.02, *R*^2^ = 24%). Adjusting for overweight or obesity did not affect the associations of AF with reduced diagnostic accuracy after HUD alone but explained more of the variance after autoEF (*P* = 0.007, *R*^2^ = 15%).

COPD was numerically and negatively associated with the GPs’ diagnostic accuracy at all diagnostic steps but did not reach statistical significance (*P* ≥ 0.24).

Neither BMI dichotomized using ≥25 and ≥30 mg/kg^2^ as thresholds nor BMI analysed as a scalar parameter showed significant associations with the diagnostic accuracy (*P* ≥ 0.05).

The associations of EF subcategories classified by the reference examination with diagnostic accuracy were weak and non-significant (*P* ≥ 0.07), even though the proportion of misclassifications was higher in patients with HFpEF. The findings did not change if EF subcategories were dichotomized by thresholds >/≤40% or </≥50% (data not given). Neither AF, COPD, nor BMI was associated with the GPs’ diagnostic uncertainty (*P* ≥ 0.1). EF had a small but significant influence on the diagnostic uncertainty after telemedical support (*P* = 0.02, *R*^2^ = 9%).

### Operators and timing

**Figure 2:**
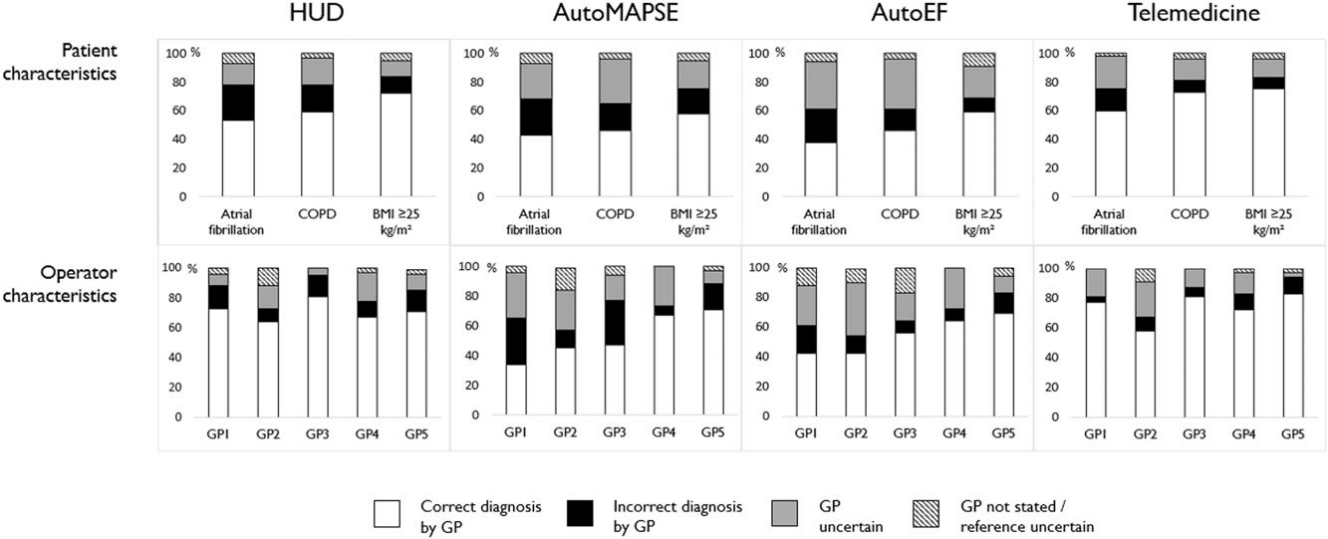
The distribution of correct, incorrect, uncertain, and not stated diagnoses according to patient characteristics and operators. The upper panel shows the distribution of correct, incorrect, uncertain, and not stated diagnoses by HUD without user support, supported by autoMAPSE, autoEF, and telemedicine according to patient characteristics, while the lower panel shows the distribution according to operators. For each column, correctly identified patients are shown in white boxes, incorrect diagnoses are shown in black boxes, cases where the GPs were uncertain of the diagnosis are shown in grey boxes, and cases where the reference cardiologists were uncertain or the HUD operators did not report an evaluation are shown at the top as not stated diagnoses.

*Figure 2* shows the distribution of correctly diagnosed patients based on patient and operator characteristics. Each of the GPs examined between 26 and 36 patients. Despite a minor numerical improvement, no significant change in the diagnostic accuracy from the first to the second half of inclusions was found (*P* ≥ 0.2). The diagnostic uncertainty after telemedical support was reduced in the second half with 7 (8%) vs. 17 (20%) of uncertain cases in the second vs. the first half, *P* = 0.03 (*Table 4*). Operator characteristics explained some of the variances in diagnostic accuracy after adding autoMAPSE (*P* ≤ 0.03, *R*^2^ = 15%), while no significant differences were found between the GPs after the other diagnostic steps, *P* ≥ 0.4 (*Table 5*). The association of operator characteristics with diagnostic accuracy using autoMAPSE was stronger and explained more of the variance when adjusted for ongoing AF [*P* ≤ 0.04, *R*^2^ = 19% (data not presented)]. Adjustments for COPD, BMI, EF, and HF did not alter these results.

**Table 4:** Operator-specific diagnostic accuracy for the first vs. the second half of inclusions by GPs

	HUD alone	AutoMAPSE	AutoEF	Telemedicine
Operator 1—first/second half of inclusions				
Correct	11 (65)/10 (63)	7 (41)/8 (50)	6 (35)/8 (50)	9 (53)/10 (63)
Incorrect	1 (6)/2 (13)	1 (6)/3 (19)	1 (6)/3 (19)	0 (0)/3 (19)
Uncertain	1 (6)/4 (25)	4 (24)/5 (31)	7 (54)/5 (31)	5 (29)/3 (19)
Operator 2—first/second half of inclusions				
Correct	6 (55)/13 (87)	3 (27)/6 (40)	3 (27)/8 (53)	6 (55)/14 (93)
Incorrect	3 (27)/1 (7)	4 (36)/(27)	4 (36)/1 (7)	1 (9)/0 (0)
Uncertain	1 (9)/1 (7)	3 (27)/5 (33)	3 (27)/(27)	4 (36)/1 (7)
Operator 3—first/second half of inclusions				
Correct	14 (78)/15 (83)	8 (44)/9 (50)	11 (56)/9 (50)	13 (72)/16 (89)
Incorrect	3 (17)/2 (12)	4 (22)/7 (39)	1 (6)/2 (11)	1 (6)/1 (6)
Uncertain	1 (6)/1 (6)	4 (22)/2 (12)	3 (17)/4 (22)	4 (22)/1 (6)
Operator 4—first/second half of inclusions				
Correct	13 (65)/11 (69)	13 (65)/11 (69)	12 (60)/11 (69)	14 (70)/12 (75)
Incorrect	3 (15)/1 (6)	1 (5)/1 (6)	2 (10)/1 (6)	3 (15)/1 (6)
Uncertain	4 (20)/3 (19)	6 (30)/4 (25)	6 (30)/4 (25)	3 (15)/2 (13)
Operator 5—first/second half of inclusions				
Correct	10 (59)/15 (83)	10 (59)/15 (83)	10 (59)/14 (78)	12 (71)/17 (94)
Incorrect	3 (17)/2 (11)	4 (24)/2 (11)	3 (17)/2 (11)	3 (17)/1 (6)
Uncertain	3 (17)/1 (6)	2 (12)/1 (6)	2 (12)/2 (11)	1 (59)/0 (0)

Data are given in numbers (percentages) for the first/second half of inclusions per operator. Uncertain represents the cases where the GPs were uncertain about the diagnosis.

**Table 5:** The influence of operator characteristics and image quality on diagnostic accuracy

	HUD alone *β* (*R*^2^)	AutoMAPSE *β* (*R*^2^)	AutoEF *β* (*R*^2^)	Telemedicine *β* (*R*^2^)
Operators				
Specific operator	−0.4 to −0.2 (0)	0.1–2.4 (15)^a^	0.5–1.2 (5)	0.0–1.8 (3)
Timing (first vs. second half)	0.7 (2)	−0.2 (0)	0.4 (1)	0.5 (1)
Image quality				
Average score		0.3 (2)	0.5 (1)	−0.1 (0.2)
Specific scores				
i. Four-chamber view		−0.02 (0)	−0.2 (3)	−0.7 (10)^a^
ii. LV centred		−0.9 (0.3)	0.2 (1)	−0.3 (2)
iii. Misalignment apex		0.3 (3)	0.1 (1)	−0.1 (0.2)
iv. Mitral annulus assessment		0.2 (1)	−0.5 (3)	−1.4 (13)^a^
v. Segments (visible endocardium)		0.4 (5)	−0.1(1)	−0.5 (3)

Data are given as unstandardized beta coefficients (*R*^2^). Explanations as in *Table 3*.

^a^Statistically significant with *P* < 0.05.

### Image quality

**Figure 3:**
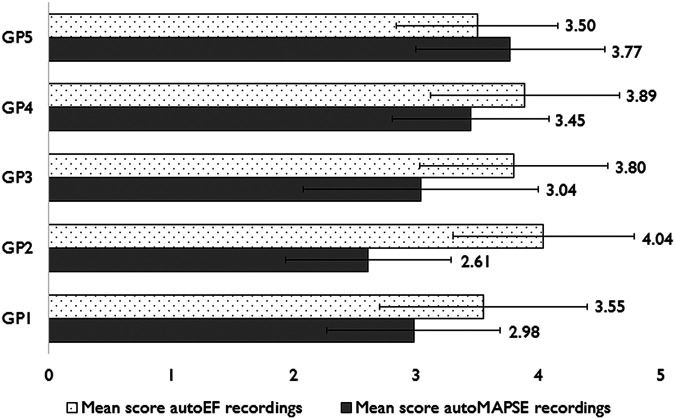
Image quality score across operators. A comparison of the mean image quality score ± SD per operator as assessed by blinded analyses in recordings acquired for analyses by automated decision-support software.

**Figure 4:**
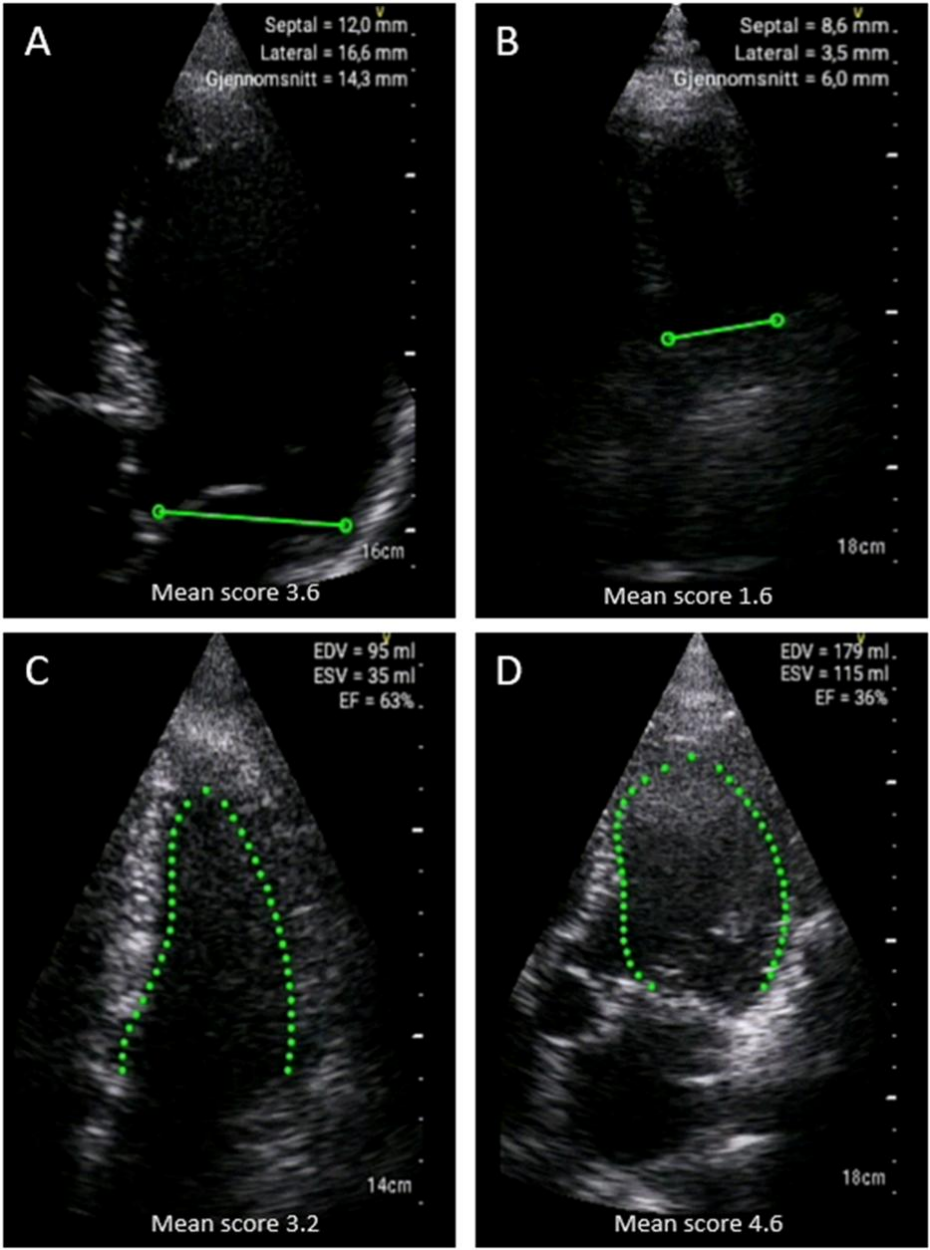
Examples of ultrasound recordings in patients with correct and incorrect diagnoses by GPs. (*A*) Correct non-HF diagnosis with normal autoMAPSE. (*B*) Incorrect HF diagnosis with reduced autoMAPSE. (*C*) Correct non-HF diagnosis with normal autoEF. (*D*) Incorrect HF diagnosis with reduced autoEF.EDV, end-diastolic volume; ESV, end-systolic volume. Explanation: ‘Gjennomsnitt’ in the upper panel is Norwegian for ‘average’.

There was a significant difference in the averaged image quality score of autoMAPSE recordings between GPs (*P* ≤ 0.003). Despite this difference, the averaged image quality score was not significantly associated with the diagnostic accuracy at any of the diagnostic steps, *P* ≥ 0.2 (*Table 5*). *Figure 3* shows the averaged image quality scores for the GPs. Lower values of image quality scores for assessments of the mitral annulus and the four-chamber view were associated with numerically reduced diagnostic accuracy at all diagnostic steps, but the associations were significant only for telemedical support (*P* < 0.05, *R*^2^ = 10–13%). *Figure 4* shows examples of correctly and incorrectly evaluated recordings.

The averaged image quality scores for autoEF recordings were similar before and after the software revision with scores of 3.9 ± 0.8 and 3.6 ± 0.6, respectively (*P* = 0.128). Additionally, the software revision did not influence the diagnostic accuracy (*P* = 0.535).

## Discussion

The diagnostic accuracy of GPs’ evaluation by HUDs was to a small degree explained by characteristics related to patients, operators, and image quality. These findings were consistent whether HUD examinations were performed as imaging interpreted by the GPs, supported by automatic LV quantification tools, or supported by telemedical expert feedback. Thus, the most important causes for the modest diagnostic accuracy were not related to factors analysed in this study even though some pre-defined image quality categories were associated with the diagnostic accuracy of the telemedical approach. The findings indicate that the technological solutions previously shown to provide a decent precision for simple tasks do not translate to the more challenging task of clinical decision-making with respect to diagnosing HF.

### Population

The prevalence of AF and COPD in the studied population was within the ranges expected for patients with suspected HF. Compared with previous studies showing a prevalence of 33–56% among HF patients,^16^ AF was present in one of four study participants and in half of the HF patients. Similarly, the proportion of the study population with HF (17%) and the proportion of HF patients with HFpEF (46%) were aligned with others.^17^ The prevalence of COPD (16%), BMI ≥25 mg/kg^2^ (80%), and obesity (36%) was aligned with current global estimates.^1,14,18^ We believe that the study population was representative of populations referred to cardiac consultations with suspected HF and that the study results can be generalized to similar populations.

### Patient characteristics

Ongoing AF was associated with reduced diagnostic accuracy for HUD examinations alone and when supported by autoEF but explained just 10% of the variance in diagnostic accuracy. After adjustment for BMI and image quality, the explained variance increased to 15–23%. In other words, despite a significant association of AF with reduced diagnostic accuracy, it only explained a minor part of the variance. The troublesome selection of which cardiac cycle to use for analyses could partly explain the results. HFpEF is known to be associated with AF and is also more challenging to diagnose.^19^ The lack of spectral Doppler and tissue Doppler modes on the HUDs adds complexity to these diagnostic procedures. The challenge was not solved by adding MAPSE and EF measurements. The low number of patients within each category could partly explain the lack of significant associations of HF categories with the diagnostic accuracy.

COPD both shares risk factors and is associated with HF. Additionally, COPD may reduce echocardiographic image quality due to a higher proportion of ultrasound wave reflections.^1,14^ In this study, COPD was numerically but not significantly associated with the GPs’ diagnostic accuracy. As the numbers of COPD patients examined by each GP were low (two to eight), these data should be carefully interpreted. Both overweight and obesity have been shown to reduce image quality.^2,18^ Despite many overweight or obese participants, BMI was not associated with the GPs’ diagnostic accuracy at any diagnostic step. Variations in fat distribution could partly explain this lack of associations.^20^ Previously, novices have been shown to identify reduced EF with a sensitivity of 65–75% irrespective of image quality^21,22^ and with significant improvements when image quality was good.^21^ In this study, the influence of HF categories on the diagnostic accuracy was minimal.

### Operator characteristics

The training programme prior to the study start was in line with current recommendations and identical for all GPs.^5^ No significant difference in the diagnostic accuracy between operators was revealed, except for when the GPs were supported by adding autoMAPSE. This finding relates to significant differences in image scores between operators in autoMAPSE recordings and indicates an inverse relation of image quality with the underestimation of MAPSE.^9,11^ The lack of significant improvement in diagnostic accuracy from the first to the second half indicates that the training of GPs was adequate. Lastly, the lack of training did not explain the modest diagnostic accuracy found in the main study.^9^

### Image quality

It has been previously shown that image quality is negatively associated with the precision of standard echocardiographic measurements.^23,24^ In our study, the overall image quality score was not associated with diagnostic accuracy, but subcategories related to the four-chamber view and the mitral annular plane influenced the accuracy after telemedical support. Thus, image quality influenced the diagnostic accuracy in qualitative assessments but not in diagnostic steps aided by automatic LV quantification. This indicates that image quality alone does not explain the poor diagnostic accuracy after the automated decision-support software and suggests a reduced performance of the software used. In recent studies, we have shown a modest reliability of the same software when used by experts.^7,11^ Despite higher feasibility and image quality by experts, reduced image quality did not explain the modest diagnostic accuracy in this study.^6,7^

### Strength and limitations

The strength of our study is the comprehensive evaluation of diagnostic accuracy in a large group of patients with suspected HF and the blinded analyses of image quality. All GPs received the same amount of training, and the operators were blinded to each other’s findings.

A larger, multicentre study with more GPs and patients could provide further insight into how the studied factors influence diagnostic accuracy. However, compared with other studies, neither the included population nor the number of operators was small.^6,21,25^ Only recordings with automatic decision-support software were analysed for image quality. An analysis of all recordings could have provided a better insight into the effect of image quality on diagnostics. The lack of NT-proBNP results always available for the GPs limits their ability to optimize the diagnostic assessment.^9^ Even though NT-proBNP has been shown suboptimal to rule in HF,^17^ a knowledge of the NT-proBNP results could have resulted in an improved identification of HFpEF patients especially by the GPs.

## Conclusion

The modest level of accuracy of the diagnostic assessment of patients with suspected HF by GPs using HUDs with and without decision-support software was just to a small part explained by patient characteristics, operator differences, and image quality. Thus, most of the variabilities in diagnostic accuracy were related to other factors. The results for the automated measurement applications indicate a need for refinement of the methods before implementation into the clinic. Lastly, the negative associations of diagnostic accuracy by the telemedical approach with some image quality characteristics indicate that efforts to improve image quality for inexperienced operators may help improve diagnostic performance.

## Data Availability

The data underlying this article will be shared on reasonable request to the corresponding author.

## Lead author biography

Malgorzata Izabela Magelssen graduated from the Medical University of Warsaw, Poland, and currently serves as a resident in cardiology at the Clinic of Cardiology, St. Olavs hospital, Trondheim University Hospital, Norway. She also holds a position as a PhD candidate at the Department of Circulation and Medical Imaging, Norwegian University of Science and Technology, Trondheim, Norway. Her thesis involves support for heart failure diagnostics using handheld ultrasound devices.
